# Tryptophan Attenuates Chronic Restraint Stress-Induced Intestinal Injury Through Modulation of Intestinal Barrier Integrity and Gut Microbiota Homeostasis

**DOI:** 10.3390/nu17060975

**Published:** 2025-03-11

**Authors:** Jianhua Zheng, Tianqi Sun, Tongtong Qin, Yunpeng Wu, Wensheng Zhang, Yefeng Qiu, Jingqing Chen

**Affiliations:** Academy of Military Medical Sciences, Beijing 100193, China; zjhcbm0311@163.com (J.Z.); suntianqi1104@163.com (T.S.); qintongtong2016@163.com (T.Q.); wuyunpeng821@163.com (Y.W.); zhangwensheng2024@163.com (W.Z.)

**Keywords:** tryptophan, chronic restraint stress, intestinal stress injury, intestinal microbiota, intestinal permeability

## Abstract

**Background:** Chronic stress is associated with detrimental effects on physical health, such as chronic restraint stress (CRS), which can damage the intestinal tract. Although tryptophan has many benefits in maintaining intestinal health, the underlying mechanism of its protective effects against stress-induced intestinal injury remains unclear. **Methods:** In this study, we constructed a CRS model by using a behavioral restraint device in which mice were restrained for 6 h per day over 14 days and investigated the effects, as well as the potential mechanism of a high-tryptophan diet (0.4% tryptophan), on CRS-induced intestinal injury using scanning electron microscopy, 16S rRNA sequencing, and LC-MS. **Results:** A 0.4% tryptophan diet (fed ad libitum for 24 days) attenuated CRS-induced pathologies, including weight loss, elevated corticosterone, intestinal barrier injury, increased permeability, and epithelial apoptosis. Tryptophan modulated the gut microbiota composition in CRS-induced mice, increasing the abundance of *Bacteroidota* and decreasing the abundance of *Firmicutes*, as well as enhancing metabolic function through pathways identified by KEGG analysis. Additionally, tryptophan restored the levels of short-chain fatty acids (SCFAs), including acetic, propionic, isobutyric, butyric, and valeric acids. Correlation analyses showed interactions between tryptophan, intestinal permeability, SCFAs, and gut microbiota. **Conclusions:** Tryptophan supplementation attenuates CRS-induced intestinal injury by modulating intestinal barrier integrity and gut microbiota homeostasis, and the beneficial effects are largely associated with the SCFA-mediated regulation of intestinal permeability and microbiota-associated energy metabolism.

## 1. Introduction

Stress represents the body’s specific or non-specific response to internal and external environmental stimuli [1,2]. It plays a pivotal role in the development and progression of numerous physical and mental disorders, including functional gastrointestinal diseases, irritable bowel syndrome, anxiety, and depression [3]. Chronic stress has been identified as a significant risk factor for psychiatric disorders such as major depressive disorder (MDD) and intestinal diseases like ulcerative colitis (UC) and Crohn’s disease (CD). Clinical evidence indicates that gastrointestinal symptoms can exacerbate depression, while individuals with depression are more prone to worsened gastrointestinal disorders [4]. Moreover, an increased risk of gastrointestinal disorders has been linked to psychiatric conditions, including depression, anxiety, schizophrenia, dementia, and autism [5]. Previous studies have also established the role of stress in contributing to cardiovascular disease, brain damage, skin disorders, and gastrointestinal problems [6].

Restraint stress, first introduced by Bonfils in 1993, has been widely utilized as a model for psychological stress in numerous studies [7,8]. Chronic restraint stress (CRS) experienced over several weeks, can induce brain abnormalities, intestinal dysfunction, and ecological disturbances [5]. The disruption of the intestinal barrier may lead to increased intestinal permeability, alterations in gut microbiota composition, and heightened inflammation [9,10]. Increasing evidence suggests that restraint stress contributes to gut damage, playing a role in the development of diseases such as inflammatory bowel disease (IBD) and irritable bowel syndrome (IBS) [7]. IBD, a chronic and debilitating gastrointestinal disorder, is currently thought to arise from an overactive immune response that attacks the gastrointestinal tract, causing ulceration and inflammation. However, its exact pathogenesis remains unclear [7]. One potential mechanism involves intestinal barrier damage, where stressors may compromise the intestinal barrier, leading to adverse outcomes such as bacterial translocation and localized or systemic inflammatory responses [6,11].

Tryptophan, an essential amino acid in mammals, plays a vital role in maintaining intestinal homeostasis, which enhances the intestinal barrier and modulates the composition and metabolism of the gut microbiota. Gut microbiota, a key component of the intestinal biological barrier, interacts with the host, either directly or through metabolites, thereby preserving intestinal microecological balance [12]. Chronic stress can exacerbate the host’s inflammatory response by disrupting intestinal microecology, further amplifying inflammation [13]. Additionally, stress-induced phenotypic traits can be transmitted via the gut microbiota [14]. Tryptophan not only acts as a nutritional enhancer but also plays a critical role in sustaining intestinal immune tolerance and gut microbiota homeostasis. Moreover, both exogenous tryptophan and endogenous tryptophan metabolites—such as kynurenine (Kyn), serotonin (5-HT), and melatonin—as well as bacterial tryptophan metabolites, like indole, indolic acid, skatole, and tryptamine, exert profound effects on gut microbial composition and metabolism, the host immune system, the host–microbiome interface, and the interactions between the host immune system and gut microbiota [15,16]. Tryptophan and its metabolites play key roles in stress-related diseases, indicating their potential effect against CRS. However, the efficacy and mechanisms of tryptophan supplementation in alleviating intestinal stress injury and maintaining gut microbiota homeostasis require further investigation. The objective of the present study was to test the hypothesis that tryptophan supplementation attenuated CRS-induced intestinal injury by regulating the intestinal barrier and gut microbiota homeostasis.

## 2. Materials and Methods

### 2.1. Reagents

We obtained a one-step TUNEL apoptosis detection kit, an H&E dyeing liquid kit, and EDTA (pH 9.0) antigen repair solution (Servicebio, Wuhan, China). We obtained the Mouse Corticosterone (CORT) ELISA Kit (Elabscience, Shanghai, China), FITC—dextran (4 kD) (Sigma, Saint Louis, MI, USA); the mouse intestinal fatty acid-binding protein (FABP) ELISA Kit (CUSABIO, Wuhan, China); and all of the 11 SCFA standards (ZZ Standards Co., Ltd., Shanghai, China). We used methanol, acetonitrile, ammonium acetate, and isopropanol (Thermo-Fisher Scientific, Fair Lawn, NJ, USA). Ultrapure water was purchased (Millipore, MA, USA). We obtained Phusion^®^ High-Fidelity DNA polymerase (New England Biolabs, Inc., Hitchin, UK) and the NEBNext Ultra II DNA Library Prep Kit, Qubit@ 2.0 Fluorometer (Thermo-Fisher Scientific, Fair Lawn, NJ, USA).

### 2.2. Animals and Diets

**Figure 1:**
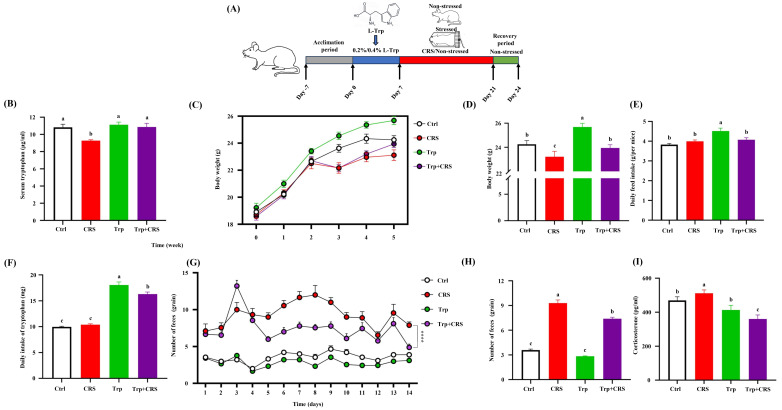
Tryptophan restored CRS-induced stress responses in the body. (**A**) A schematic diagram of the trail; (**B**) serum tryptophan content. (**C**,**D**) body weight changes; (**E**,**F**) average daily food intake and tryptophan intake; (**G**,**H**) quantity of feces and average feces production of mice 20 min after restraint stress; (**I**) serum corticosterone hormone content. Different letters indicate significant differences between groups, *p* < 0.05. **** *p* < 0.0001. Ctrl refers to the control group.

At the age of 7 weeks, sixty male BALB/c mice were purchased from Charles River (Beijing, China) and maintained in plastic cages under standard conditions with free access to feed and water. After 1 week of acclimatization, mice were randomly assigned into four groups (*n* = 9). In the control group (Ctrl), mice were fed a basal diet; in the chronic restraint stress group (CRS), mice were fed a basal diet and placed in a homemade behavioral restriction device for 6 h each day (the restraint device is a specially designed breathable 50 mL centrifuge tube with a diameter of 3.5 cm; it has four holes punched in the sides near the bottom and one hole punched in the center of the lid [17]; the pattern is shown in Figure 1A); in the tryptophan group (Trp), mice were fed a tryptophan-supplemented diet; and in the tryptophan and chronic restraint stress group (Trp + CRS), mice were fed a tryptophan-supplemented diet and placed in a homemade behavioral restriction device. Basal diets with 0.20% tryptophan and a tryptophan diet with 0.40% tryptophan were used in our study. Mice in the Trp and Trp + CRS group were supplemented with 0.4% tryptophan 1 week before the induction of CRS stress and throughout the whole trial. The amino acid content in the basal diet was determined by HPLC, as described previously [18], and is shown in Table A1. Mice in the CRS group and Trp + CRS group were placed in a homemade behavioral restriction device for 6 h each day, from 9:00 to 15:00, over a period of 14 days. Body weights, as well as food intakes, were recorded. Additionally, the fecal particles produced in the first 20 min were also recorded.

Mice were anesthetized with 1% pentobarbital sodium at a dose of 5 μL/g of body weight by intraperitoneal injection after 6 h of fasting, and then blood was collected from the retro-orbital sinus at the end of the experiment. After dissection, duodenum and colon tissues were collected, washed with pre-cooled PBS buffer, and either immediately fixed in 4% formaldehyde solution or were snap-frozen in liquid nitrogen and then stored at −80 °C until analysis.

All experimental operations were in accordance with animal welfare ethics and approved by the Committee for the Management and Use of Laboratory Animals of the Military Medical Research Institute [IACUC-DWZX-2022-049]. Mice were kept in the barrier facility environment of the Experimental Animal Center of the Military Medical Research Institute [SYXK (Military) 2017-0022] at a constant temperature of 23 ± 2 °C, a relative humidity of 40–60%, and an alternating light and dark time of 12 h/12 h, with 3 mice/cage (plastic mouse cage, L × W × H: 403 mm × 165 mm × 175 mm).

### 2.3. Analysis of Tryptophan Concentration in Serum

The use of tryptophan in serum was analyzed by HPLC according to the national standard (GB/T 30987-2020) [19]. Firstly, 0.1 mol/L hydrochloric acid solution, 50% acetonitrile solution, tryptophan standard working solution, and tryptophan standard intermediate solution were prepared. Then, the mobile phases were prepared: 200 mmol/L ammonium formate solution, 20 mmol/L ammonium formate–water solution (mobile phase A), and 20 mmol/L ammonium formate–acetonitrile solution (mobile phase B). The extract was transferred to a 10 mL volumetric flask, and then diluted by adding 50% acetonitrile solution. It was fixed to the scale and gently mixed. Then, 2 mL of the diluted solution was taken and centrifuged at 4 °C for 10 min at 14,000 r/min. Finally, the supernatant was taken and assessed by HPLC.

### 2.4. Histological Analysis

Fixed duodenal and colonic tissues were dehydrated and embedded in paraffin, and slices of 5 μm sections were collected with hematoxylin and eosin (H&E) staining to observe morphology, as previously described [20]. The stained sections were observed under a light microscope and photographed, with the length of duodenal villi and crypt depth measured using Image J 1.53t software.

Distal colons were fixed in 2.5% glutaraldehyde (Servicebio, Wuhan, China). After rinsing with PBS, the samples were fixed in 1% osmium tetroxide (prepared in PBS buffer) for 1–2 h and protected from light. Following additional PBS rinses, the tissues were dehydrated through a gradient ethanol series and replaced with isoamyl acetate, with each step lasting 15 min. The samples were then dried using a critical point dryer, mounted on double-sided adhesive with conductive carbon film, and coated with gold in an ion sputtering apparatus for approximately 30 s. Finally, villi were observed using an electron microscope.

### 2.5. ELISA

Mouse serum corticosterone content was detected using competitive ELISA according to the kit instructions. The optical density (OD) was measured at a 450 nm wavelength using an enzyme marker (detection was completed within 5 min).

### 2.6. Intestinal Permeability

Intestinal permeability was assessed using the FITC-conjugated dextran 4 kDa (FD4) metho. Mice were gavaged with a PBS solution containing FITC-dextran (4 kDa, Sigma, Saint Louis, MI, USA) at a concentration of 40 mg/mL, with each mouse receiving a dose of approximately 100 µL per 10 g of body weight [20]. Three hours after gavage, 100 µL serum was collected, and the fluorescence intensity was detected at an excitation wavelength of 485 nm using a multi-plate reader (SpectraMax iD3, Molecular Devices, San Jose, CA, USA). The serum FITC-dextran concentration was calculated from a standard curve for serially diluted FITC-dextran.

Additionally, serum intestinal fatty-acid binding protein (IFABP), another intestinal permeability indicator, was also detected, and this was conducted according to the kit’s instructions. The optical density (OD) was measured at a wavelength of 450 nm using a multi-plate reader (SpectraMax ABS plus, Molecular Devices, San Jose, CA, USA).

### 2.7. Tunnel Staining

TUNEL apoptosis detection was performed according to the manufacturer’s instructions. Briefly, sections of the duodenum were deparaffinized, treated with proteinase K at 37 °C for 22 min, subjected to 0.1% Triton X-100 (Servicebio, Wuhan, China) for 20 min, and incubated with TdT enzyme solution and a fluorescent labeling solution at 37 °C for 2 h. Nuclei were re-stained with DAPI (1:1000 dilution), and apoptosis-positive cells were observed by a blinded observer under a fluorescence microscope and photographed using a Nikon imaging system DS-U3. Apoptotic cells were quantified by randomly counting 8 different microscopic fields for each section.

### 2.8. Quantitative Real-Time PCR

Total RNA was extracted and then reverse-transcribed into cDNA, as instructed by the manufacturer. Real-time PCR was performed using the SYBR Premix Ex Taq II (TaKaRa, Kusatsu, Shiga, Japan) and the CFX96 Touch Real-Time PCR Detection System (Bio-Rad, Hercules, CA, USA) according to the manufacturers’ instructions. Table A2 lists the primers used in the present study. *β-actin* was used as a reference gene for normalization and the 2^−ΔΔCt^ method was used to determine the fold change in the mRNA level.

### 2.9. 16S Sequencing Analysis

The total genomic DNA of fecal bacteria was extracted by using a DNA Kit (Qiagen, Hilden, Hesse, Germany) according to the manufacturer’s instructions. The integrity of DNA was assessed by agarose gel electrophoresis, and then the genomic DNA was used as a template for PCR amplification. The 16S RNA V3–V4 gene region was amplified by using the primers F515 and R806. PCR amplification was conducted using a prepared fusion primer reaction solution, utilizing the previously prepared DNA. The 16S rRNA gene was sequenced on the Illumina Novaseq6000 sequencing platform at the Novogene Co., Ltd. (Beijing, China) according to the manufacturer’s instructions.

The QIIME2 process was used to analyze alpha diversity and beta diversity, and the pictures were drawn with the R (v4.0.3) package. All the sequencing data were submitted to the National Center for Biotechnology Information GenBank Sequence Read Archive database under accession number PRJNA1222112 (https://www.ncbi.nlm.nih.gov/bioproject/PRJNA1222112, accessed on 11 February 2025).

### 2.10. Measurement of Short-Chain Fatty Acids

An ultra-high performance liquid chromatograph coupled to a tandem mass spectrometry (UHPLC-MS/MS) system (Vanquish^TM^ Flex UHPLC-TSQ Altis^TM^, Thermo Scientific Corp, Braunschweig, Germany) was used to quantitate SCFAs in Novogene Co., Ltd. (Beijing, China). Separation was performed on a Waters ACQUITY UPLC BEH C18 column (2.1 × 100 mm, 1.7 μm), which was maintained at 40 °C. The mobile phase, consisting of 10 mM ammonium acetate in water (solvent A) and acetonitrile: isopropanol (1:1) (solvent B) was delivered at a flow rate of 0.30 mL/min. The solvent gradient was set as follows: initial 25% B, 2.5 min; 25–30% B, 3 min; 30–35% B, 3.5 min; 35–38% B, 4 min; 38–40% B, 4.5 min; 40–45% B, 5.5 min; 45–50% B, 5.5 min; 50–55% B, 6.5 min; 55–58% B, 7 min; 58–70% B, 7.5 min; 70–100% B, 7.8 min; 100–25% B, 10.l min; 25% B, 12 min. The mass spectrometer was operated in a negative multiple reaction mode (MRM). Parameters were set as follows: IonSpray Voltage (−4500 V), Sheath Gas (35 psi), Ion Source Temp (550 °C), Auxiliary Gas (50 psi), and Collision Gas (55 psi).

### 2.11. Statistical Analyses

All data are presented as means ± SEM. Statistical analysis was performed using SPSS 26.0 software. For comparative data analysis between groups, we applied one-way ANOVA and Duncan’s multiple range test. For 16S rRNA gene sequencing data, α diversity (Chao1, goods_coverage, Shannon, and Simpson index) was assessed by QIIME2. β diversity was calculated based on unweighted unifrac distances by QIIME2. An unweighted unifrac PCoA was performed based on ASVs to provide an overview of the microbial diversity in and composition of the mouse feces. Other graphs were generated using GraphPad Prism 9.0 software and R (v4.0.3) package. A significance level of *p* < 0.05 was applied for all analyses. Spearman correlation coefficents (*p* < 0.05) and heatmaps were utilized to disclose the correlation between tryptophan, intestinal permeability, SCFAs, and gut microbiota via R (v4.0.3) software.

## 3. Results

### 3.1. Tryptophan Restored CRS-Induced Stress Responses in the Body

Compared with the control group, the concentration of Trp in the serum of CRS-induced mice decreased (*p* < 0.05), whereas it significantly increased in the Trp + CRS group in response to dietary Trp supplementation (Figure 1B). CRS significantly decreased the body weights and feed intakes (*p* < 0.05), which were mitigated by tryptophan supplementation, leading to faster weight recovery post-stress (*p* < 0.05) (Figure 1C,D) and higher daily tryptophan intake (Figure 1E,F).

Within 20 min of CRS, in mice, feces numbers significantly increased, while they were suppressed after tryptophan administration (*p* < 0.05) (Figure 1G,H). Furthermore, CRS elevated corticosterone levels, an effect also mitigated by tryptophan supplementation (*p* < 0.05) (Figure 1I), indicating a beneficial effect on body stress.

### 3.2. Tryptophan Attenuates CRS-Induced Intestinal Injury

**Figure 2:**
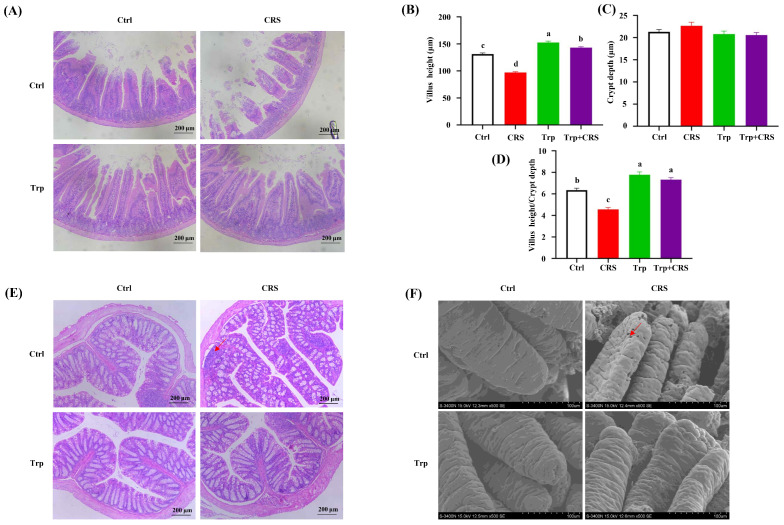
Tryptophan attenuates CRS-induced intestinal injury. (**A**) Duodenal histopathology (H&E); (**B**–**D**) duodenal villus length, crypt depth, and villus length/crypt depth values; (**E**) colon histopathology (H&E), arrow indications point to inflammatory cell infiltration accompanied by markedly abnormal crypt structures; (**F**) scanning electron microscopy of duodenal tissue, arrows indicate damaged duodenal villi with increased permeability; different letters indicate significant differences between groups, *p* < 0.05. Ctrl refers to control group.

To further investigate the effects of tryptophan on the intestinal barrier function in mice subjected to CRS, we observed intestinal morphology. As shown in Figure 2, mice supplemented with tryptophan were significantly protected against intestinal injury by CRS. Compared with mice fed on a basal diet, tryptophan-fed mice exhibited a milder disease process with a significant reduction in intestinal villi shortening, inflammatory cell infiltration, and intestinal wall thinning (*p* < 0.05) (Figure 2A–E). Furthermore, the smooth, well-arranged, and robust duodenal villi were atrophied and widened by CRS, the damage from which was markedly mitigated by tryptophan supplementation (Figure 2F), indicating its noticeable protective effect on impaired morphology.

### 3.3. Tryptophan Attenuates CRS-Induced Intestine Leakage and Epithelial Apoptosis

**Figure 3:**
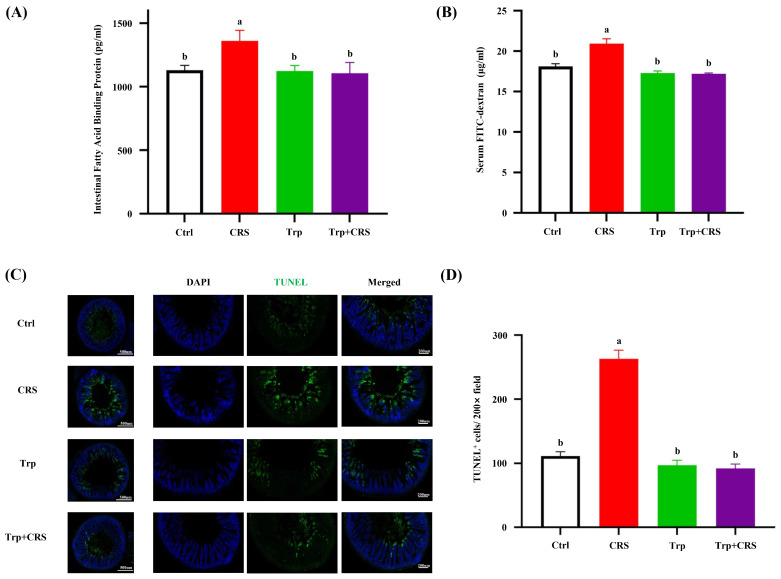
Tryptophan alleviates CRS-induced intestine leakage and epithelial apoptosis. (**A**) Serum intestinal fatty acid-binding protein content by ELISA; (**B**) FITC-dextran content in serum; (**C**) TUNEL staining plots; (**D**) TUNEL-positive cell counts; different letters denote significant differences between groups, *p* < 0.05. Ctrl refers to control group.

To further assess the impact of tryptophan on intestinal injury under restraint stress conditions, we also assessed intestinal permeability using the permeability markers intestinal fatty acid-binding protein (I-FABP) and FITC-dextran. The results showed that tryptophan supplementation significantly lowered serum I-FABP levels and FITC-dextran contents in chronically restrained mice (*p* < 0.05) (Figure 3A,B), indicating the significant alleviation of stress-induced increases in intestinal permeability. Additionally, a measurable increase in intestine apoptosis (Figure 1I) was observed in chronically restrained mice (*p* < 0.05), and this was significantly mitigated by tryptophan supplementation (Figure 3C,D).

### 3.4. Tryptophan Activates the Expression of Intestinal Kyn and 5-HT Metabolism-Related Enzyme Genes

**Figure 4:**
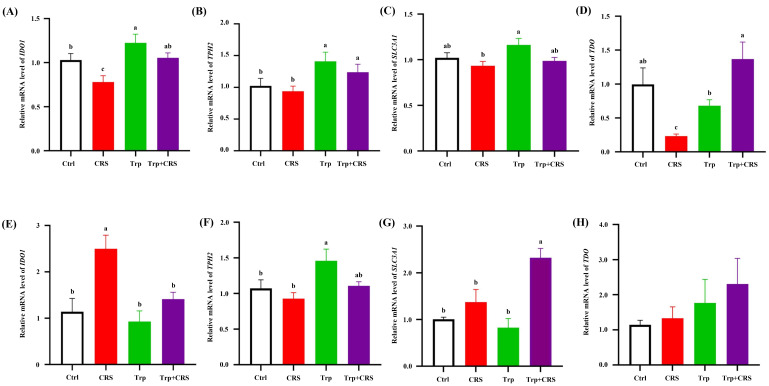
Tryptophan activates the expression of intestinal Kyn and 5-HT metabolism-related enzyme genes. (**A**–**D**) Relative expression of *IDO1*, *TPH2*, *SLC3A1*, and *TDO* mRNA levels in duodenal tissues; (**E**–**H**) relative expression of *IDO1*, *TPH2*, *SLC3A1*, and *TDO* mRNA levels in colonic tissues; different letters denote significant differences between groups, *p* < 0.05. *β-Actin* was used as an internal reference and Ctrl refers to the control group.

Tryptophan can enhance the barrier function through its metabolites such as Kyn, KYNA, and 5-HT. The expression of genes related to the metabolic pathways of Kyn and 5-HT in the intestines was detected. The gene expression of *IDO1*, *TPH2*, and *SLC3A1* in the duodenum, as well as that of *TPH2*, and *SLC3A1* in the colon, was significantly increased (*p* < 0.05), (Figure 4A–H) by dietary tryptophan supplementation under CRS, indicating the activation of intestinal tryptophan, Kyn, and 5-HT metabolism.

### 3.5. Tryptophan Inhibits CRS-Induced Gut Microbiota Dysfunction

**Figure 5:**
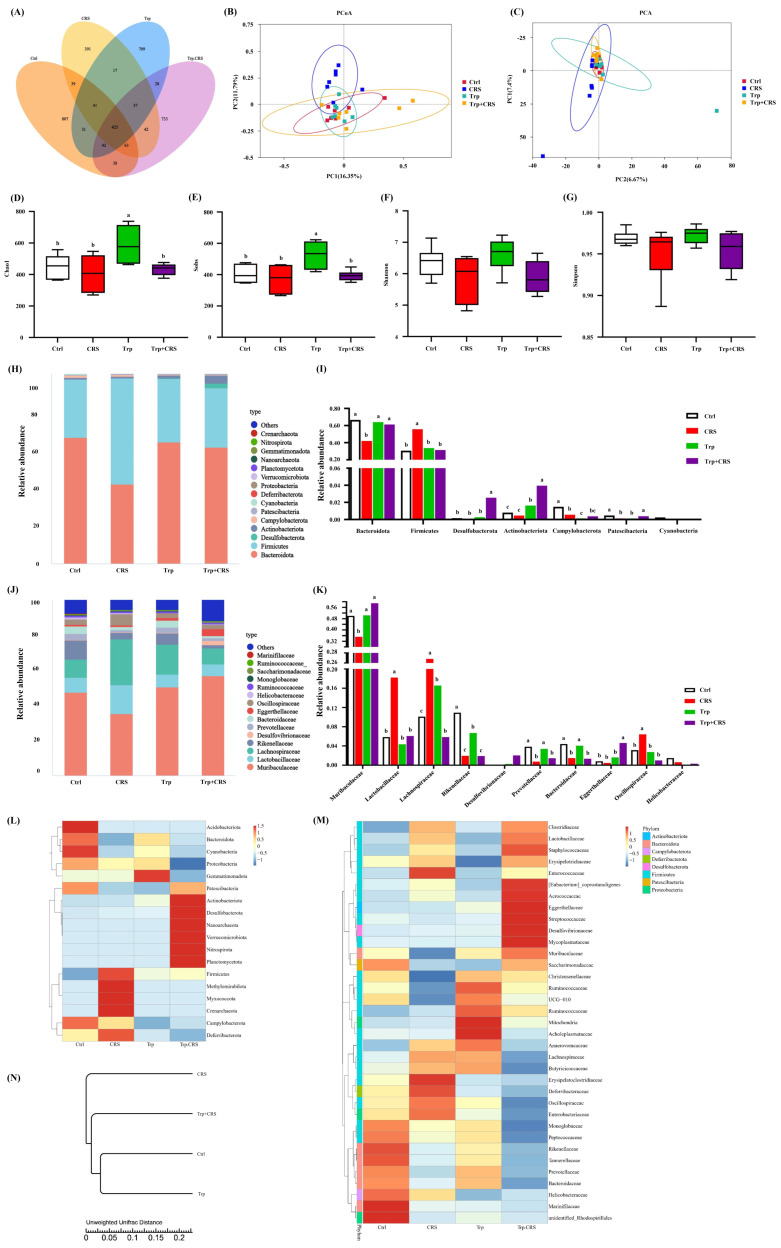
Tryptophan inhibits CRS-induced gut microbiota dysfunction. (**A**) Venn diagram distribution of ASVs between groups; (**B**) PCoA analysis; (**C**) PCA analysis; (**D**–**G**) alpha diversity Chao1, Sobs, Shannon, and Simpson indices, in that order; (**H**,**I**) phylum-level colony abundance maps, and the relative abundance of dominant bacterial taxa; (**J**,**K**) phylum- and family-level species clustering heat maps; (**L**,**M**) section-level microbiota abundance map and the relative abundance of dominant bacterial taxa; (**N**) Unifrak hierarchical clustering tree; different letters indicate significant differences between groups, *p* < 0.05. Ctrl refers to the control group.

**Figure A1:**
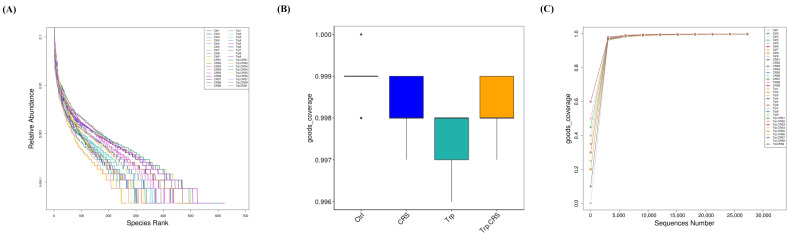
Analysis of microbial diversity and community structure: (**A**) species rank-abundance curves; (**B**) boxplot of Good’s coverage estimator for different groups; (**C**) rarefaction curves for each group.

High-throughput sequencing of 16S rRNA gene amplicons was performed to assess the effect of tryptophan on CRS-induced gut microbiota dysfunction. Good’s coverage and rarefaction curves indicated that the sequencing depth was sufficient to capture the majority of microbial communities in the samples (Figure A1A–C). Compared with the CRS group, the mice in Trp + CRS group showed significantly increased α-diversity metrics for Chao1 and Sobs indices (*p* < 0.05), but not Simpson and Shannon indices (Figure 5A,D–G), in response to dietary supplementation with Trp. Unweighted principal coordinate analysis (PCoA) and principal component analysis (PCA) also indicated a noticeable difference between CRS and Trp + CRS mice using β-diversity analysis (Figure 5B,C), indicating the modulation of gut microbiota homeostasis by tryptophan supplementation under restraint conditions. In terms of differences in the abundance and dominant species of gut microbiota, at the phylum level, tryptophan supplementation mainly affected the relative abundance of *Bacteroidota*, *Firmicutes*, *Actinobacteriota*, *Campylobacterota*, and *Patescibacteria* (Figure 5H). The abundance of *Bacteroidota*, *Actinobacteriota*, and *Campylobacterota* decreased, while the level of *Firmicutes* increased in CRS mice, and tryptophan supplementation suppressed these CRS-induced changes (Figure 5I). At the family level, tryptophan affected *Muribaculaceae*, *Lactobacillaceae*, *Lachnospiraceae*, *Rikenellaceae*, *Prevotellaceae*, *Bacteroidaceae*, *Eggerthellaceae*, *Oscillospiraceae*, and *Helicobacteraceae* in terms of relative abundance (Figure 5J). In CRS mice, the presence of *Muribaculaceae* and *Actinobacteriota* decreased, the relative abundance of mice in the CRS group was significantly decreased, and the levels of *Lactobacillaceae*, *Lachnospiraceae*, and *Oscillospiraceae* increased. Tryptophan supplementation mitigated these changes (Figure 5K) and suggested a healthier microbial profile. Additionally, *Rikenellaceae*, *Prevotellaceae*, and *Bacteroidaceae* decreased in CRS mice and were further affected by tryptophan.

To assess microbial species distribution, we constructed a species clustering heatmap and a weighted UniFrac hierarchical clustering tree. The heatmap revealed that the gut microbiota structures of the control and tryptophan groups were similar, while the CRS group differed significantly from the others (Figure 5L,M). The clustering tree confirmed these findings, showing structural similarity between the control and tryptophan groups. CRS treatment reduced *Proteobacteria* abundance and increased the *Firmicutes*/*Bacteroidota* ratio, markers of intestinal homeostasis that were attenuated by tryptophan supplementation.

### 3.6. Prediction Analysis of Tryptophan for the Metabolic Pathways of CRS-Induced Microbiota

**Figure 6:**
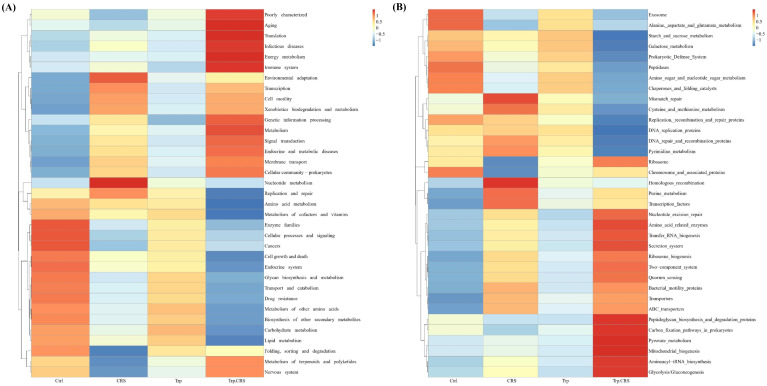
Prediction analysis of tryptophan on the metabolic pathways of CRS-induced microbiota. (**A**,**B**) Tax4Fun functional annotation of possible functional pathways at different levels of gut microbiota and their metabolic functions based on KEGG database.

To investigate the contribution of gut microbiota to host metabolism, correlation prediction analysis of metabolic pathways was conducted. Working based on amino acid, carbohydrate, energy, and lipid metabolism, membrane transport, translation, replication, and repair information processing, it can be stated that a total of 18 pathways were significantly affected by dietary tryptophan under restraint stress (Figure 6A,B), indicating the potential ability of tryptophan to attenuate gut stress injury by modulating the gut microbiota and enhancing the above metabolic pathways.

### 3.7. Tryptophan Inhibits CRS-Induced Decrease in SCFAs

**Figure 7:**
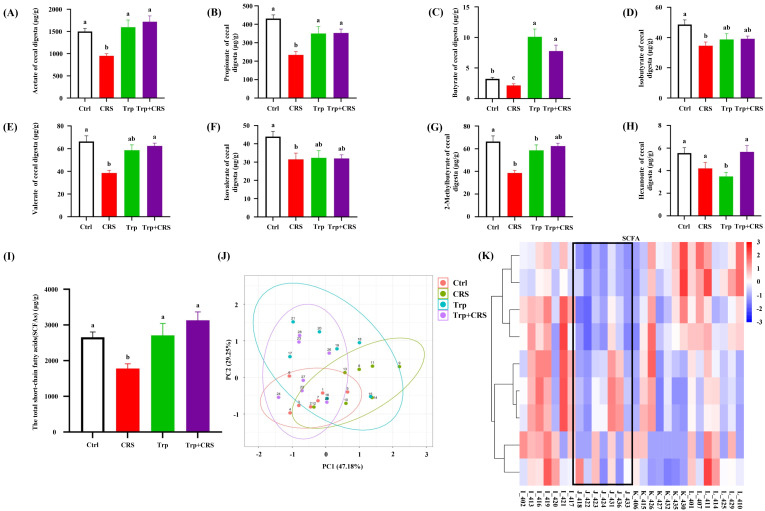
Tryptophan inhibits CRS-induced decreases in SCFAs. (**A**–**H**) The concentration of SCFAs in the cecum, in order of the acetic acid, propionic acid, butyric acid, isobutyric acid, valeric acid, isovaleric acid, 2-methylbutyric acid, and hexanoic acid content of the cecum; (**I**) total SCFA content; (**J**) PCA analysis; (**K**) cluster analysis plot; different letters indicate significant differences between different groups, *p* < 0.05. Ctrl refers to the control group.

To explore whether the changes in the intestinal microbiota affect the production of SCFAs following Trp supplementation, the SCFAs of the hindgut contents were determined using LC-MS. Elevations in concentrations of acetic, propionic, butyric, valeric, and 2-methylpentanoic acid, and in the total SCFAs levels, were observed in the Trp + CRS group relative to the CRS group (*p* < 0.05) (Figure 7A–I). Moreover, PCA and clustering analyses showed that tryptophan significantly altered the metabolic profile of SCFAs in CRS mice (Figure 7J,K). All these data suggest that dietary tryptophan affects the production of SCFAs in CRS mice.

### 3.8. Correlations Between Tryptophan, Intestinal Permeability, SCFAs and Gut Microbiota

**Figure 8:**
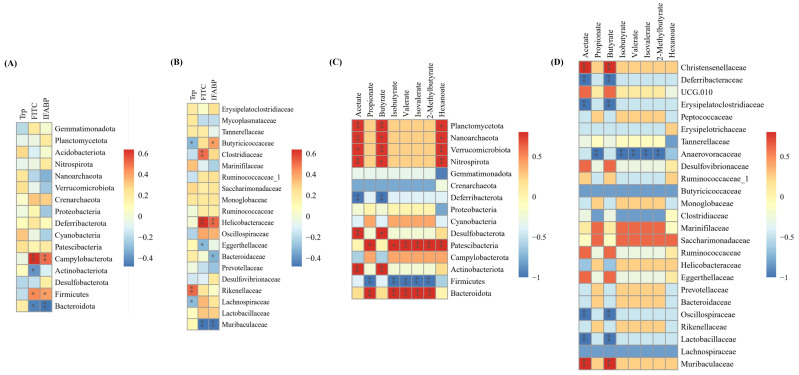
Correlations between tryptophan, intestinal permeability, SCFAs, and gut microbiota. (**A**,**B**) Heat maps of serum tryptophan, intestinal permeability, and gut microbiota-related networks, in order of gate and family level; (**C**,**D**) heat maps of SCFAs and gut microbiota-related networks, in order of gate and family level. * *p* < 0.05, ** *p* < 0.01.

To further explore the potential interactions between tryptophan and the gut microbial composition, as well as metabolism, in CRS-induced mice, the tryptophan, intestinal permeability indicators, gut microbes, and their metabolites SCFAs were selected to calculate the Spearman correlation coefficients. This was visualized in a clustered heat map (Figure 8). Notably, the serum tryptophan content positively was correlated with bacterial species from the genera *Cyanobacteria* and *Rikenellaceae*. In addition, there was a significant correlation between *Firmicutes* and *Campylobacterota* (the phylum level), *Helicobacteraceae* and *Clostridiaceae* (the family level), and indicators of intestinal permeability evaluation (e.g., IFABP, FITC-dextran) (Figure 8A,B). Most SCFAs (such as acetic, propionic, butyric, valeric, 2-methylpentanoic acid) are positively correlated with bacterial species from the genera *Bacteroidota*, *Patescibacteria*, *Actinobacteriota*, *Desulfobacterota* (the family level), *Muribaculaceae*, *Saccharimonadaceae*, *Marinifilaceae*, and *Christensenellaceae* (the family level) (Figure 8C,D). These data suggest that tryptophan is closely associated with gut microbes and their metabolites, SCFAs, and may play a role by modulating the microbes and metabolites involved.

## 4. Discussion

In the present study, we found that tryptophan supplementation attenuates CRS-induced intestinal injury. This beneficial effect is associated with the modulation of intestinal barrier integrity and gut microbiota homeostasis.

Stress is implicated in the pathogenesis of gastrointestinal disorders, including IBD and IBS, by altering brain-gut interactions and disrupting intestinal barrier function [21,22,23,24]. Environmental factors, including restraint stress, are key triggers for IBD episodes [23]. In mice, restraint stress disrupts the intestinal barrier and gut microbiota metabolic function, triggering inflammation, impairing intestinal barrier integrity, and increasing permeability [10,25,26]. Consistent with previous findings, CRS in this study induced intestinal injury by impairing morphology, altering permeability, and disrupting intestinal barrier integrity. Tryptophan alleviates stress and improves intestinal health, although the exact mechanisms remain incompletely understood.

Tryptophan, an essential amino acid that can be easily extracted and which plays a vital biological role, offers therapeutic potential for preventing and treating CRS-induced toxicity and associated damage [27,28]. Dietary tryptophan supplementation and tryptophan-rich substances can enhance stress and anxiety resistance in both humans and animals [29,30]. As the primary site of nutrient absorption, intestine injury impairs nutrient digestion and absorption [31], thus affecting host health. In this study, tryptophan alleviated CRS-induced weight loss, elevated serum corticosterone levels, and intestine damage. Koopmans et al. demonstrated that tryptophan supplementation significantly increased the conversion of 5-hydroxytryptophan (5-HTP) into serotonin (5-HT) in the hypothalamus of stressed piglets and reduced salivary corticosterone levels, indicating its ability to suppress stress responses [32]. This may represent a key mechanism by which tryptophan attenuates the CRS-induced elevation of serum corticosterone levels.

Prolonged stress disrupts gastrointestinal function and increases intestinal permeability [6,8,33]. Apoptosis, a key mechanism of barrier dysfunction, induces epithelial damage and immune dysregulation [34]. It has been shown that tryptophan may also promote intestinal health by improving antioxidant status and reducing inflammation, ER stress, apoptosis, and pyroptosis in piglets following lipopolysaccharide challenges [35]. In the present study, tryptophan alleviated CRS-induced intestinal leakage and decreased apoptosis, as evidenced by its inhibition of IFABP, FITC-dextran, and TUNEL-positive cell levels.

Stress or immunological challenges can disrupt tryptophan metabolic pathways in the brain and intestinal tissues [29,30]. The kynurenine (Kyn) and serotonin (5-HT) metabolic pathways of tryptophan may play key roles in the initiation and progression of stress-induced diseases [28]. In animals, endogenous tryptophan metabolism primarily follows the Kyn and 5-HT pathways. The Kyn pathway regulates inflammation, metabolism, immune responses, and neurological function, while the 5-HT pathway modulates intestinal homeostasis, sleep, and gut–brain axis signaling [28]. Key enzymes in these pathways include IDO and TPH, respectively [36,37]. Additionally, TDO and IDO are critical rate-limiting enzymes in the Kyn pathway, and SLC3A1 facilitates the transmembrane transport of large neutral amino acids, such as tryptophan. The dysregulation of SLC3A1 may impair amino acid transport [28]. The abnormal expression of *TPH2* and *IDO1* has been linked to 5-HT system dysfunction and mental disorders like depression, whereas upregulating TPH2 and IDO1 enhances tryptophan metabolism, exerting antidepressant-like effects [38]. In this study, tryptophan supplementation significantly increased the gene expression of *IDO1*, *TPH2*, and *SLC3A1* in the duodenal tissues, as well as increasing *TPH2* and *SLC3A1* in the colonic tissues, of CRS-exposed mice, indicating the important role of tryptophan in metabolic pathways under stress conditions.

Tryptophan also plays an important role in maintaining intestinal homeostasis by regulating the composition and metabolism of the intestinal microbiota. The intestinal microbiota is a key component of the intestinal biobarrier and interacts with the host, either directly or through metabolites, thereby maintaining intestinal microecological balance [12,39]. In the present study, tryptophan mitigated CRS-induced gut microbiota dysfunction, as shown by changes in α-diversity and relative abundance. The phyla *Bacteroidota* and *Firmicutes* were the most abundant in the gut microbiota across all groups, with *Firmicutes* being more prevalent in the CRS group. After tryptophan supplementation, we saw increased *Bacteroidota* and decreased *Firmicutes*, suggesting a healthier microbial profile. This can potentially reduce inflammation and enhance barrier function. While the *Bacteroidota*/*Firmicutes* ratio did not differ among the control, tryptophan, and Trp + CRS groups, functional prediction analysis using the KEGG database indicated the enhancement of metabolic activity in tryptophan-supplemented groups. Tryptophan supplementation in European sea bass regulates metabolic responses, particularly in energy metabolism and stress reactions, mitigating acute stress effects from immune challenges under restricted conditions [40]. These findings suggest that tryptophan altered the gut microbiota structure and promoted the growth of beneficial bacteria in CRS mice, and the precise mechanisms behind this require further investigation.

SCFAs, gut-derived microbial metabolites, possess immunomodulatory and anti-inflammatory properties that are crucial for maintaining intestinal homeostasis and regulating immune responses [41]. These beneficial effects are mediated through two primary mechanisms—the induction of immune tolerance and the enhancement of intestinal barrier integrity—both of which are influenced by gut microbial composition [41]. It has been shown that tryptophan significantly increases the production of SCFAs by bacteria, including *Clostridium*, unclassified bacteria, and *Vibrio acetobacter* spp. [42]. Among the SCFAs, acetic and propionic acids constitute the major fermentation products, followed by butyric, isovaleric, valeric, and isobutyric acids [43]. These metabolites maintain intestinal barrier function by upregulating tight junction (TJ) proteins and modulating gut microbiota–immune cell interactions [44]. Our findings demonstrate that tryptophan supplementation effectively reversed the CRS-induced impairment of gut microbiota metabolic functions, particularly in SCFA production, during which the concentrations of acetic acid, propionic acid, butyric acid, valeric acid, 2-methylbutyric acid, and total SCFA levels were markedly decreased. These results indicate that the potential role of tryptophan in stress-related gut microbiota dysfunction and microbial metabolite change.

Correlation analyses confirmed that tryptophan supplementation significantly modulates gut microbiota composition, potentially exerting its functions by altering the interactions among SCFAs, intestinal permeability, and gut microbiota. The comparative analysis of energy and metabolism-related gene expression revealed that tryptophan’s protective effects against CRS-induced intestinal injury are mediated through the SCFA-dependent regulation of intestinal permeability and gut microbiota-associated energy metabolism. We identified significant correlations between specific microbial taxa (*Bacteroidota*, *Patescibacteria*, *Actinobacteriota*, and *Desulfobacterota*), both in terms of SCFAs levels and intestinal permeability markers. The increased abundance of *Muribaculaceae*, *Bacteroidaceae*, and *Prevotellaceae* may enhance intestinal barrier function, reduce intestinal permeability, and attenuate inflammatory responses by modulating metabolites such as short-chain fatty acids and bile acids [45,46,47]. Following tryptophan supplementation, the rise in these beneficial bacteria may further regulate host energy metabolism and neuroendocrine function through their metabolites, thereby maintaining intestinal homeostasis [48,49]. The findings indicate that gut microbiota may maintain intestinal barrier integrity through SCFAs production, which promotes epithelial repair and regeneration [49,50]. This mechanistic insight explains how tryptophan ameliorates CRS-induced gut dysbiosis and facilitates recovery from intestinal stress injury.

## 5. Conclusions

In conclusion, in the present study, we found that tryptophan administration attenuates CRS-induced intestinal injury, as evidenced by the reduction in stress hormones, intestinal histopathological injury, intestine leakage, and epithelial apoptosis, as well as the modulation of gut microbiota composition and SCFAs production. The effects of tryptophan on intestinal barrier integrity and gut microbiota homeostasis were associated via the SCFA-mediated regulation of intestinal permeability and microbiota-associated energy metabolism. Considering the high incidence of stress-related diseases (IBD, IBS, UC), and the impairment of intestinal barrier function in CRS, tryptophan supplementation may represent a potential adjuvant therapy capable of maintaining intestinal integrity under stress conditions. More studies are needed to investigate how gut dysbiosis is alleviated and how tryptophan contributes to maintaining the intestinal barrier’s integrity, function, and health.

## Data Availability

The datasets generated during and analyzed during the current study are available from the corresponding authors upon reasonable request due to time limitations.

## Appendix A. Amino Acid Content and Gene Expression Analysis

### Appendix A.1. Amino Acid Content in Diets with Varying Tryptophan Levels

L-tryptophan (with a purity greater than 98%) was added to the basal feed to produce supplemental tryptophan feed. We determined the content of amino acid in the basal feed as well as in the supplemental tryptophan feed for mice. The tryptophan content in the basal feed for this experiment was 0.20%, and the content in the supplemental tryptophan feed was 0.40%. Details are provided in the table below:

**Table A1:** Amino acid content in diets with varying tryptophan levels.

Ingredient	Basic Feeds	Supplemental Tryptophan Feeds
Glycine	0.96%	0.90%
Alanine	0.96%	0.92%
Serine	0.87%	0.82%
Proline	1.38%	1.28%
Valine	0.90%	0.85%
Serine	0.74%	0.70%
Isoleucine	0.75%	0.70%
Leucine	1.49%	1.41%
Asparagine	1.83%	1.71%
Lysine	1.06%	0.96%
Glutamate	3.70%	3.46%
Methionine	0.20%	0.23%
Histidine	0.57%	0.51%
Phenylalanine	0.93%	0.87%
Arginine	1.37%	1.21%
Serine	0.61%	0.54%
Cysteine	0.25%	0.26%
Total 17 amino acids	18.57%	17.33%
Tryptophan	0.20%	0.40%

### Appendix A.2. Sequence List of qRT-PCR Primers

Table A2 provides a detailed list of gRT-PCR primer sequences used for gene expression analysis. The table includes forward and reverse primers for five genes: *β-Actin*, *SLC3A1*, *IDO1*, *TDO*, and *TPH2*.

**Table A2:** Sequence list of qRT-PCR primers.

Gene	Forward Primer (5′ → 3′)	Reverse Primer (5′ → 3′)
*β-Actin*	GGCTGTATTCCCCTCCATCG	CCAGTTGGTAACAATGCCATGT
*SLC3A1*	ATGAAGGGATGCCGAACCAAT	CAGGGATACTCACGGCGTTG
*IDO1*	GCCTCCTATTCTGTCTTATGCAG	ATACAGTGGGGATTGCTTTGATT
*TDO*	ATGAGTGGGTGCCCGTTTG	GGCTCTGTTTACACCAGTTTGAG
*TPH2*	GGTTGTCCTTGGATTCTGCTG	GCCTGGATTCGATATGAAGCAT

## Appendix B. Analysis of Microbial Diversity and Community Structure

Sparsity curves demonstrated high sample coverage and species richness, with nearly 99% Good’s coverage across all four sample sets, indicating that the sequencing depth was sufficient to capture the majority of microbial communities in the samples. Figure A1 illustrates microbial diversity and community structure across different groups.
